# Development of a Promising Method for Producing Oligomeric Mixture of Branched Alkylene Guanidines to Improve Substance Quality and Evaluate Their Antiviral Activity against SARS-CoV-2

**DOI:** 10.3390/molecules26113472

**Published:** 2021-06-07

**Authors:** Denis O. Shatalov, Stanislav A. Kedik, Ivan S. Ivanov, Anna V. Aydakova, Diana A. Akhmedova, Dmitrii S. Minenkov, Sergei V. Beliakov, Alexander Herbst, Lasse Greiner, Liubov I. Kozlovskaya, Viktor P. Volok

**Affiliations:** 1 Institute of Fine Chemical Technologies Named after Lomonosov, MIREA—Russian Technological University, 119571 Moscow, Russia; shat-05@mail.ru (D.O.S.); doctorkedik@yandex.ru (S.A.K.); ivan.ivanov1994@gmail.com (I.S.I.); ann.reznikova2012@ya.ru (A.V.A.); s.v_beliakov@mail.ru (S.V.B.); 2 Institute of Pharmaceutical Technology, 119571 Moscow, Russia; 3 Ishlinsky Institute for Problems in Mechanics RAS, 119526 Moscow, Russia; minenkov.ds@gmail.com; 4 Wingflow AG, Gaensaker 5, 5070 Frick, Switzerland; herbst@wingflow.ch; 5 Mannheim University of Applied Sciences, 68163 Mannheim, Germany; greiner@wingflow.ch; 6 FSBSI “Chumakov FSC R&D IBP RAS”, 108819 Moscow, Russia; lubov_i_k@mail.ru (L.I.K.); viktor.p.v@mail.ru (V.P.V.); 7 Institute of Translational Medicine and Biotechnology, Sechenov Moscow State Medical University, 117418 Moscow, Russia

**Keywords:** antiseptic, microfluidics, microreactor, polycondensation, antimicrobial resistance, antiviral activity

## Abstract

This paper reports the synthesis of branched alkylene guanidines using microfluidic technologies. We describe the preparation of guanidine derivatives at lower temperatures, and with significantly less time than that required in the previously applicable method. Furthermore, the use of microfluidics allows the attainment of high-purity products with a low residual monomer content, which can expand the range of applications of this class of compounds. For all the samples obtained, the molecular-weight characteristics are calculated, based on which the optimal condensation conditions are established. Additionally, in this work, the antiviral activity of the alkylene guanidine salt against the SARS-CoV-2 virus is confirmed.

## 1. Introduction

Diseases caused by various infections are considered to be among the most common pathologies that a person acquires in the modern world. The optimism associated with the synthesis of a new class of drugs (antibiotics) at the beginning and middle of the 20th century was extinguished by the development of antimicrobial resistance (AMR) due to the overuse of these drugs. Annually, at least 50,000 people die from infectious diseases caused by drug-resistant microorganisms in Europe and the USA, and in developing countries, this number reaches hundreds of thousands [1]. These infectious diseases include pneumonia, tuberculosis, human immunodeficiency virus, malaria, oncology, against which antibiotics play a decisive role, particularly in chemotherapy and surgical procedures in general (e.g., organ transplantation and caesarean section). The World Health Organization has adopted the Global Strategy for the Containment of Antimicrobial Resistance, which includes various approaches aimed at guaranteeing the effectiveness of vital drugs, such as antibiotics [2]. Without concerted action, many antimicrobial agents (AMAs) can lose their potency because of the increase in AMR not only in the current generation, but also in the future. According to experts [3], in the chemotherapy of infectious diseases, it is permissible to use several AMAs, e.g., a combination of antibiotics and antiseptics. This is because the microbial association has different levels of sensitivity, and the combination of AMAs enables the prevention or retardation of the AMR formation in pathogens and enhances their inactivating effect based on the synergistic activity of the active substances. Thus, the principle of combining AMAs targets both indifference (the independence of the separate effects of two AMAs when combined) and summation (the combined effect of the two AMAs, equivalent to the sum of their separate effects). However, AMR can also be acquired against antiseptics, which is expressed as the loss of sensitivity to the minimum inhibitory concentration and contact time.

At present, arguably no bacteria resistance has been detected for drugs that include polyguanidines; therefore, such drugs can be recommended for use in medical practice together with antibiotics [4]. A study showed their effectiveness as sporicidal agents for combating bacterial spores and nosocomial (nosocomial) infections [5,6,7,8,9,10,11]. In addition, polyguanidines can be included in, for example, dressings for the treatment of chronic wounds [12]. Quite recently, it was found that low-molecular-weight guanidines (molecular weight: 800 ± 200 Da), called oligoguanidines, exhibit improved bactericidal, antiviral and antifungal activities compared to the embodiments of the closest analogue, which are also associated with their tendency to branch [13]. Furthermore, given the epidemiological situation caused by the outbreak of COVID-19, it is relevant to assess the effect of active pharmaceutical ingredients (APIs) on SARS-CoV-2 [14,15]. The use of polyguanidines and oligoguanidines as APIs is severely limited by the high content of impurities and the anisotropy of their molecular-weight characteristics, which are associated with their production. The conventional methods have several disadvantages, among which are the excessive content of residual monomers in the final product and the anisotropy of the physicochemical properties of the reaction mass. The heterogeneity of the molecular-mass characteristics cannot be sufficiently eliminated by mixing; therefore, the effective polycondensation rate constants can vary significantly depending on the volume of the reaction mass and time differently when heating is applied. This leads to a deterioration in the reproducibility of the yield, and the spread of the molecular characteristics and biological activity of the product over a wide range. In addition, upscaling the process, including the mixing stage, requires revealing and considering the complex relationships between the reactor geometry and the mixing device, as well as the intensity of mixing with the hydrodynamic regime and heat transfer during heating. The development of new synthesis methods that can eliminate these shortcomings will significantly expand the range of use of guanidine-type antiseptics.

A rapidly developing area in the field of chemical synthesis is microfluidics: an interdisciplinary field of research that studies methods for monitoring and controlling fluid flows on a micron scale, i.e., in a microreactor [16]. The use of microreactors allows us to solve the anisotropy problem, since the gradients of temperature and concentration of the reagents and products in the direction perpendicular to their flow are negligible because of the small diameter of the microreactor; additionally, at a low linear velocity, these gradients are insignificant in the direction of flow. The literature does not contain data on the microfluidic synthesis of alkylene guanidines or related compounds, which indicates that any research in this area would be innovative and pioneering, giving scientists room to focus on reducing the economic costs of the process. It was found that polycondensation with the formation of branched alkylene guanidine can be effectively conducted at a low optimum temperature [17,18]. Thus, during preliminary tests, it was found that the supply of reagents or their mixtures to the microreactor in the form of concentrated aqueous solutions allows the polycondensation process to be conducted stably, i.e., with a small number of technological failures [19]. Taking into account a need for the synthesis of new antimicrobial agents on one hand, and the development of a promising method for producing them on the other, the aim of this work was to develop a microreactor method for producing branched oligoguanidines with a narrow molecular-weight distribution. The molecular-weight characteristics, which were used to determine the optimal condensation conditions (time, temperature, and the ratio of the initial reagents), were calculated using the ^13^C-NMR Spectroscopy. Based on the obtained data, the parameters of the microfluidic synthesis process were optimized. Additionally, in this work, the antiviral activity of the alkylene guanidine salt synthesized by the microfluidic method against the SARS-CoV-2 virus was investigated.

## 2. Materials and Methods

### 2.1. Preparation of the Reaction Mixture and Microreactor Synthesis

Aqueous solutions with different component ratios were prepared using guanidine hydrochloride (GH) (Sigma-Aldrich, Saint Louis, MO, USA) and hexamethylenediamine (HMDA) (Acros Organics, Geel, Belgium) under physicochemical conditions, according to Table 1.

**Figure 1:**
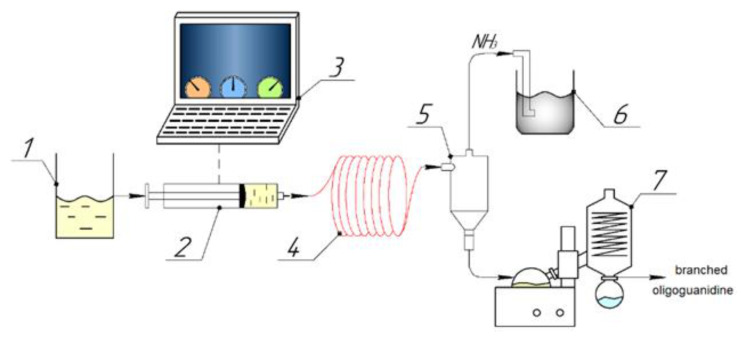
This is a figure. Schemes follow the same formatting. 1—GH and HMDA aqueous solution; 2—medium-pressure syringe pump module; 3—PC; 4—microreactor; 5—cyclone; 6—flask-receiver of ammonia; 7—rotary evaporator.

To supply the reaction mixture to the microreactor module with the possibility of separate temperature control, a high-precision medium-pressure syringe pump module with a capillary diameter of 1/8 inch (Qmix Pro Ext company of Wingflow AG, Frick AG, Switzerland) with check valves was used to generate continuous flows (neMESYS MPM, Qmix Pro Ext company of Wingflow AG, Frick AG, Switzerland), as shown in Figure 1.

The temperature and flow rate were controlled using a PC with the QmixElements software preinstalled (Qmix Pro Ext company of Wingflow AG). A gas separator (cyclone) (Wingflow, AG, Frick, Switzerland) was used to remove ammonia. After synthesis in the microreactor, the mixture containing the target product was evaporated on an RV 10 rotary evaporator (IKA, Staufen, Germany). The purification of the starting monomers and low-molecular-weight fraction was carried out by reprecipitation in a water–acetone mixture according to the following procedure: a 10% intermediate product obtained by the microreactor method was prepared in a round-bottom flask. Thereafter, acetone was added to the prepared solution in the amount necessary to cloud the solution, followed by separation. The mixture was kept until a distinguishable interface appeared, after which the upper transparent layer was decanted, and the contents of the lower layer were evaporated.

**Figure 2:**
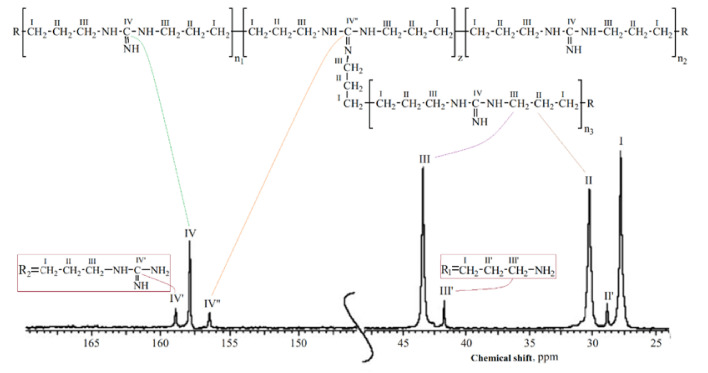
Typical ^13^C-NMR spectrum of OHMG-HC.

A typical nuclear magnetic resonance (NMR) spectrum of OHMG–HC is shown in Figure 2.

The ^13^C-NMR spectra of the samples (D_2_O, 300 MHz) were recorded using a DPX NMR spectrometer (Bruker, DPX, Karlsruhe, Germany). Chemical shifts were reported in units of δ (ppm) relative to tetramethylsilane. The number of branches per molecule (z) was calculated based on the integral signal intensities of the ‘unbranched’ and ‘branched’ units and the end fragments of GH and HMDA, and it is expressed as follows in Equation (1):

(1) $$z=\frac{2-\frac{2b}{a+1}}{\frac{2b}{d}+3b+\frac{b}{a+1}-1}$$

where coefficients *a*, *b* and *d* are expressed in terms of the integral signal intensities of S_II_, S_III_, S_IV_, S_II′_, S_III′_, S_IV′_ and S_IV″_, corresponding to the carbon atoms, as follows in Equations (2)–(4):

(2) $$a=\frac{S_{\mathrm{III}}\prime }{S_{\mathrm{IV}}\prime }$$

(3) $$b=\frac{S_{\mathrm{III}\prime }+S_{\mathrm{IV}}\prime }{S_{\mathrm{III}}}$$

(4) $$d=\frac{S_{\mathrm{IV}}\prime \prime }{S_{\mathrm{IV}}}$$

The number average molecular weight (M_n_) of the sample was calculated based on the number of ‘unbranched’ and ‘branched’ units, end fragments of GH and HMDA, and their molecular weights (141, 182, 100 and 58, respectively), and it is represented by Equation (5).

(5) $$M_{n}=\frac{z}{d}*141+z*182+\frac{2+z}{a+1}*100+a*\frac{2+z}{a+1}*58$$

### 2.2. ESI-TOF MS Analysis

ESI-TOF mass spectrum was acquired using a micrOTOF-Q II (Bruker Daltonics, Bremen, Germany) mass spectrometer equipped with electrospray ionization. A sample solution was prepared using CH_3_CN as solvent. Ions were generated by electrospray ionization below 120 °C. The results were analyzed with Compass Data Analysis 4.0 SP 5 software.

### 2.3. High-Performance Liquid Chromatography Analysis

For the determination of the HMDA and GH contents in the substance, high-performance liquid chromatography (HPLC, Thermo Fisher Scientific, San Jose, CA, USA) was employed. The gradient method was used for both analyses.

HMDA was determined using the Luna C18(2) 250 × 4.6 mm (5 µm) column (Phenomenex, Inc., Torrance, CA, USA) at 30 °C and 264 nm. Mobile phase A is comprised of MilliQ water; mobile phase B is comprised of acetonitrile (HPLC grade). The gradient program is presented in Table 2.

For GH determination, the Luna C18(2) 150 × 4.6 mm (5 µm) column (Phenomenex) was used. The analysis was carried out at 25 °C and 205 nm. Mobile phase A is comprised of 0.087% sodium 1-pentanesulfonate in 1% aqueous solution of orthophosphoric acid; mobile phase B is comprised of acetonitrile (HPLC grade). The gradient program is presented in Table 3.

### 2.4. Investigation of the Antiviral Activity of the Drug Against SARS-CoV-2

In the experiments, we used Vero (green monkey kidney) cell line (RCB 10-87, WHO, Switzerland). Cells were maintained in 2×EMEM medium (Eagle Minimum Essential Medium with doubled amino acids and vitamins), supplemented with 5% fetal bovine serum (FBS) (Gibco, Thermo Fisher Scientific, Inc., Waltham, MA, USA), streptomycin (0.1 mg/mL), and penicillin (100 units/mL) (PanEco, Moscow, Russia).

SARS-CoV-2 strain PIK35 (GISAID ID EPI_ISL_428852) was isolated from a nasopharyngeal swab of a COVID-19 patient [20]. The virus was passaged 5 times in Vero cells and stored as an infected cells suspension at −80 °C.

The antiviral activity was assessed in titer reduction assay, i.e., the inhibitory effect was estimated as compound concentration decreasing viral titer by 50%.

Vero cells were seeded in 96-well plates (approximately 10^5^ cells per well) and incubated at 37 °C in a CO_2_ incubator for 3 days until a full monolayer was formed. A compound was added to the virus suspension (1000 TCID_50_) in four concentrations, starting from 5.6 mM. The resulting mixtures were incubated for 1 h at room temperature, then the remaining infectious virus was quantified via titration in Vero cells by its ability to cause cytopathic effect (CPE). The virus titers were calculated according to the Karber method [21]. The antiviral effect was determined by the decrease in the titer of the virus in the samples with the compound in comparison with the negative control (cell culture medium) samples. The EC_50_ was calculated using the approximation method.

## 3. Results

### 3.1. Analysis of the Spectra

**Figure 3:**
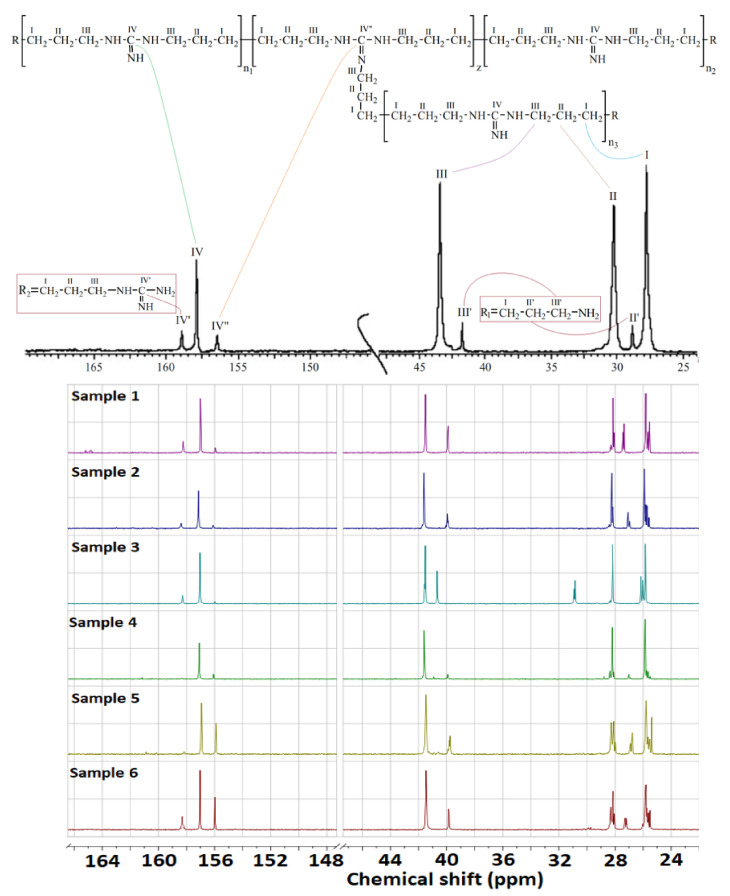
^13^C-NMR spectra of the obtained samples.

The results of the ^13^C-NMR spectroscopy analysis enabled the identification of the characteristic functional groups, as shown in Figure 3.

The authenticity of the synthesized compounds was confirmed by comparing the signals in a typical NMR spectrum with those in the spectra of the obtained samples (Table 4), based on which the spectrum of sample 6 was chosen as the most consistent.

From the obtained spectra of each of the samples, M_n_ and z were calculated (Table 5), and the residual impurities were analyzed. Based on the analysis, we established that the M_n_ increases and the z value decreases as the amount of the initial HMDA content decreases, which was confirmed by the results of syntheses 1–4. In syntheses 5 and 6, on the contrary, a strictly opposite picture was observed. The purest product was obtained by the synthesis of series 5 and 6, with a reagent ratio of GH/HMDA = 1/1. The spectral convergence of sample 6 confirmed that it contained the lowest number of impurities.

### 3.2. Optimisation of Parameters

Here, the goal is to define the parameter values that give the desired characteristics of the resulting oligomer (M_n_ = 800, and z = 0.4). Firstly, the comparison results of batches 1 with 2 and 3 with 4 show that their temperature dependence is weak; thus, it is sufficient to consider only batches 2, 4, 5 and 6 with a temperature of 160 °C. Secondly, the approximations of M_n_ and z, as functions of L and h, were obtained, using dependences with the simplest necessary nonlinearity in the following form (6) and (7):

Mn (L, h) = m_1_ + m_2_h + m_3_L + m_4_hL (6)

z (L, h) = c_1_ + c_2_h + c_3_L + c_4_hL (7)

Coefficients M_i_ and C_i_ are obtained from linear algebraic systems (8):

M_n_ (L_j_, h_j_) = M_nj_, z (L_j_, h_j_) = z_j_, (8)

j = 2, 4, 5, 6 is the batch number.

**Figure 4:**
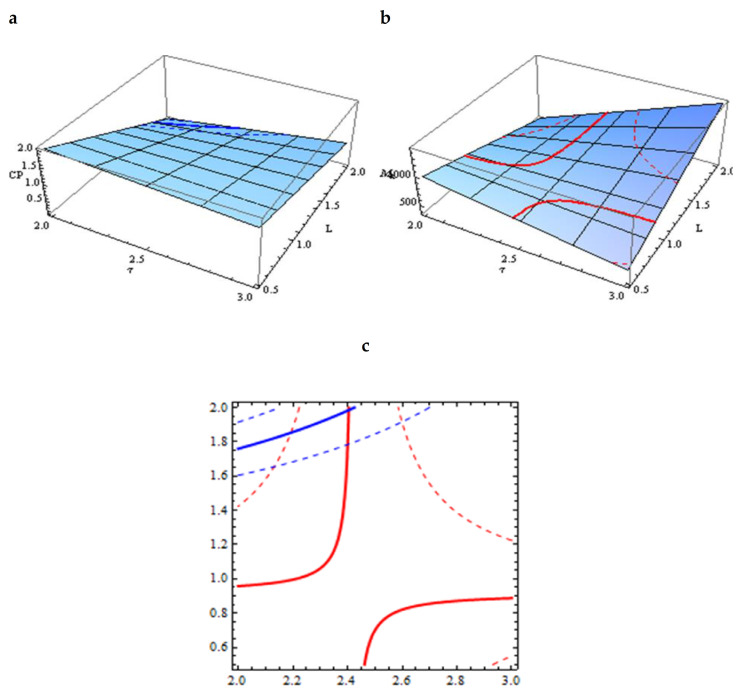
Approximations of Mn (L, h) and z (L, h) based on batches 2, 4, 5 and 6. (**a**) z as functions of L and h; (**b**) Mn, as functions of L and h; (**c**) Overlay plots of Mn and z, as functions of L and h.

The determinant of both systems is the same and equals 1, which implies the existence of a unique solution, M_i_ and C_i_ (Table 6). The corresponding approximations are shown in Figure 4.

Finally, using the obtained approximations, the optimal values of L = 2 (i.e., 1/0.5) and h = 2.4 (i.e., 2 h 24 min), at T = 160 °C, were calculated to obtain the required oligomer characteristics (M_n_ = 800, and z = 0.4).

### 3.3. Verification

**Figure 5:**
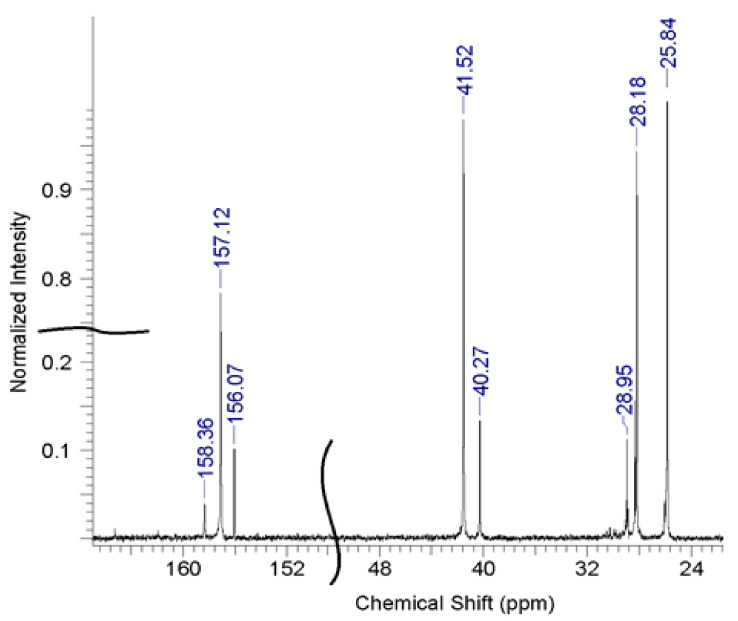
NMR spectrum after optimization.

**Figure 6:**
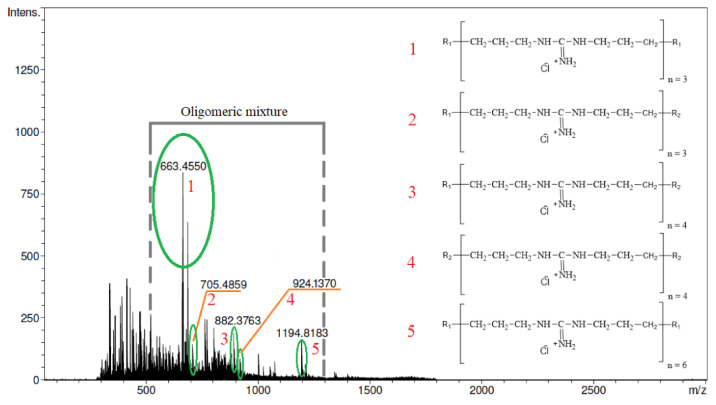
ESI-TOF mass spectrum after optimization.

To verify the prediction made using approximations and optimizations, a new series of experiments were conducted for L = 1/0.5, h = 2 h 24 min, and T = 160 °C. The obtained results match the requirements with good precision (Table 7 and Figure 5 and Figure 6).

### 3.4. Verification of the Antiviral Activity

OHMG-HC activity was assessed in vitro by its ability to decrease SARS-CoV-2 infectivity, i.e., its ability to infect cells. Due to high toxicity of the compound, titer decreasing assay was chosen as a method of choice. The assay was calibrated using N-hydroxycytidine (NHC), an established inhibitor of SARS-CoV-2 reproduction [22,23]. OHMG-HC decreased the SARS-CoV-2 titers in a dose-dependent manner, i.e., the inhibition efficiency increased with the drug concentration. The 50% effective concentration (EC_50_) values are presented in Table 8. OHMG-HC showed comparatively higher activity against SARS-CoV-2 than NHC.

## 4. Discussion

Based on the results of this work, we can conclude that the principles of microfluidics can be successfully applied to guanidine-type antiseptic production. The advantages of flow systems can solve the problems associated with the anisotropy of the molecular-mass characteristics of the obtained substance and reduce the economic costs of its production, as evidenced by the low synthesis temperature and reduced time.

Compared to traditional synthesis, where the process proceeds at temperatures up to 200 °C for up to 7.5 h, the flow-through technology facilitates efficient polycondensation at 160 °C for 3 h, which reduces the economic costs of obtaining the product.

Another important advantage of the proposed technology is the low number of impurities contained in the final compound, as well as the corresponding content of chlorides in the final product; in this regard, the quality of the substance increases.

Additionally, a study on the antiviral activity of OHMG–HC showed that it works against the SARS-CoV-2 virus, which is important considering the current epidemiological situation.

All the above may serve as a basis for expanding the applicability of branched oligoguanidines in widespread medical practice.

## Figures and Tables

**Table 1:** Parameters of the microreactor synthesis.

Batch	GH/HMDA Ratio, L	Temperature, °C	Residence Time, h
1	1/0.5	150	2
2	1/0.5	160	2
3	1/0.5	150	3
4	1/0.5	160	3
5	1/1	160	2
6	1/1	160	3

**Table 2:** Gradient Program for the HPLC analysis of HMDA.

Time, min	Phase A,%	Phase B,%
0	60	40
1	60	40
10	10	90
16	10	90
17	60	40
20	60	40

**Table 3:** Gradient Program for the HPLC Analysis of GH.

Time, min	Phase A,%	Phase B,%
0	100	0
3	100	0
4	10	90
15	10	90
16	100	0
30	100	0

**Table 4:** The correlation of the signals of a typical NMR spectrum and the spectrum of Sample 6.

Atom Designation	δ (Standard), ppm	δ (Sample 6), ppm
IV′	157.11	158.25
IV	156.08	157.01
IV″	154.61	155.96
III	41.72	41.45
III′	40.10	39.81
II	28.56	28.14
II′	27.60	27.28
I	26.09	25.81

**Table 5:** Molecular-mass characteristics of the Samples and the impurity content.

Batch	M_n_	Z	GH, wt%	HMDA, wt%
1	307	0.04	0.121	0.032
2	426	0.14	0.137	0.048
3	1498	0.79	0.529	0.084
4	1421	0.85	0.523	0.082
5	782	1.38	0.324	0.058
6	867	1.41	0.339	0.062

**Table 6:** Approximation of coefficients M_i_ and C_i_.

I	1	2	3
M_i_	3083	−936	−2471
C_i_	4	−0.7	−2.7

**Table 7:** Molecular-mass characteristics of the Samples and the impurity content for the defined optimal parameters (l = 1/0.5, h = 2 h 24 min and t = 160 °C).

Batch	M_n_	Z	GH, wt%	HMDA, wt%
1	786	0.38	0.231	0.012
2	814	0.37	0.233	0.013
3	802	0.38	0.232	0.011
4	793	0.38	0.231	0.011

**Table 8:** Antiviral activity of the GH/HMDA in Vero cells against SARS-CoV-2.

Compound	ED_50_, µM (Mean ± SD)
OHMG-HC	60 ± 22
NHC	438 ± 10

## Data Availability

Data is contained within the article.

## Sample Availability

Not available.
